# Prepublication Communication of Research Results

**DOI:** 10.1007/s10393-018-1352-3

**Published:** 2018-08-07

**Authors:** Michael J. Adams, Reid N. Harris, Evan H. C. Grant, Matthew J. Gray, M. Camille Hopkins, Samuel A. Iverson, Robert Likens, Mark Mandica, Deanna H. Olson, Alex Shepack, Hardin Waddle

**Affiliations:** 1 Forest and Rangeland Ecosystem Science Center, U.S. Geological Survey, 3200 SW Jefferson Way, Corvallis, OR 97330 USA; 2 Department of Biology, MSC 7801, James Madison University, Harrisonburg, USA; 3 Patuxent Wildlife Research Center and SO Conte Anadromous Fish Laboratory, U.S. Geological Survey, Laurel, USA; 4 Center for Wildlife Health, University of Tennessee Institute of Agriculture, Knoxville, USA; 5 U.S. Geological Survey, Reston, USA; 6 Wildlife Health Section, Environment and Climate Change, Canadian Wildlife Service, Gatineau, Canada; 7 Pet Industry Joint Advisory Council, Alexandria, USA; 8 The Amphibian Foundation, Atlanta, USA; 9 U.S. Forest Service, Corvallis, USA; 10 Department of Biological Sciences, Florida International University, Miami, USA; 11 Wetland and Aquatic Research Center, U.S. Geological Survey, Gainseville, USA

Publishing of scientific findings is central to the scientific process, and it is traditional to consider findings “provisional” until accepted by a peer-reviewed journal. Until publication, communication of provisional findings beyond participants in the study is typically limited. This practice helps assure scientific integrity. However, a dilemma arises when a provisional finding has urgent societal consequences that may be exacerbated by delay. This dilemma may be particularly pronounced when a discovery concerns wildlife health, which could have implications for conservation, public health (i.e., zoonoses), or domestic animal health (e.g., avian influenza). A scientist may see a need for prepublication communication but consider such communication to be problematic. We suggest that common concerns about directed prepublication communication are generally misplaced. Our perspective comes from natural resources science and management, but we suspect that this situation could arise in any branch of science and that discussing these issues will help scientists who may not routinely work with public officials navigate an unfamiliar situation.

Our collective experience suggests that communication of unpublished results is generally limited to conversations among close colleagues or presentations at scientific conferences where there is a mutual understanding, often emphasized during presentations, that unpublished findings are provisional. Scientists can be more reluctant to communicate directly with public officials who might make decisions based on provisional findings. This restraint is born out of caution and respect for the scientific process that relies on peer review to catch errors and temper conclusions. It is also sometimes meant to avoid disclosures of important results to the media before publication in a journal because such disclosure may be seen as unethical or it may be perceived to reduce options for future publication.

A precarious situation arises when a finding has critical time-sensitive implications like the discovery of an emerging pathogen in a novel region. This came to our attention because of a situation involving the potential discovery of the amphibian fungal pathogen commonly called Bsal (short for *Batrachochytrium salamandrivorans*) that is currently causing salamander die-offs in Europe (Stegen et al. 2017). Bsal is not known to occur in North America (AmphibiaWeb 2018), but laboratory studies suggest that North American amphibians are susceptible (Martel et al. 2013; Stegen et al. 2017) and the risk to native amphibians is high (Yap et al. 2015; Richgels et al. 2016). Efforts have been made to prevent the introduction of Bsal to the USA (Fish and Wildlife Service 2016) and Canada (Canada Border Services Agency 2017) but concerns remain, and there are extensive preparations under way for the expected discovery of Bsal (Grant et al. 2016). Select communication of a provisional discovery of Bsal cannot await the normal scientific review.

A Bsal Task Force was formed in 2015 to coordinate North American research, surveillance, and other preparation needed to respond to a novel finding of Bsal (see salamanderfungus.org). The Task Force has explored various discovery and response scenarios, and part of that discussion has highlighted concerns harbored by some scientists about prepublication communication of scientific findings that might delay communication of the provisional finding to public officials who could be acting to limit its effects. Any delay in communication could have dire repercussions for amphibians in North America which are already experiencing major declines (Adams et al. 2013).

Our experience suggests that the concerns about communicating a provisional finding to public officials fall into five categories: (1) employer policy; (2) uncertainty in the finding; (3) jeopardizing manuscript acceptance by a journal; (4) ethics; and (5) fear of being “scooped.” Our contention is that these concerns largely stem from misperceptions and that none should cause a delay in the communication of time-sensitive provisional findings to appropriate authorities.

*Employer policy* Research institutions may have policies regarding the review and dissemination of scientific findings that are meant to safeguard their credibility and to prevent scientists from running afoul of regulations related to their conduct. US government agencies have such policies, but the primary US federal agencies involved in natural resources research can and do communicate findings to authorities prior to publication in a journal under certain circumstances. Not all scientists have experience with this situation, and some may not realize that early communication is routine and expected with any findings that have important and immediate consequences. Scientists can work with their institutions to navigate this process.

*Uncertainty in the finding* There is always some amount of uncertainty in a scientific finding. For example, a laboratory test might indicate the presence of genetic material for a pathogen of interest but that result is not 100% convincing that the pathogen is actually present. False positives can result from contamination or a lack of specificity in the test (Iwanowicz et al. 2017) and are of particular concern when searching for a pathogen in a region where it has not been previously detected. There can be concern among scientists that public officials will not adequately incorporate uncertainty into their decision making. This might be especially problematic when a finding is likely to be sensational or have serious implications. Communicating provisional information opens the scientist to the possibility of appearing to “jump the gun” and a small possibility of eventually needing to publically change or withdraw the finding. We urge scientists to trust public officials such as natural resource managers with important but provisional information. In our experience, such individuals are skilled at addressing uncertainty and at managing sensitive preliminary information judiciously and confidentially until it is published. Indeed, response plans often address levels of uncertainty directly (Grant et al. 2017).

*Jeopardizing acceptance by a journal* Does communicating a finding with public officials break the embargo policy of journals or constitute prior publication? What if the finding leaks to the press? What if the public officials do a press release as part of their management response? The answer is that none of these situations jeopardize publication in a journal including high-profile journals. To verify this, we queried editors at Nature, PLoS One, PLoS Pathogens, Diseases of Aquatic Organisms, Herpetological Review, Frontiers in Ecology and Evolution, and Science. We received responses from all except PLoS Pathogens and Science. Editors confirmed that even a press release by public officials would not alter their review process or jeopardize publication. While scientists generally should not publicly release findings prior to publication in a journal, they are free to communicate with appropriate officials even if those officials deem it necessary to communicate more broadly with the public.

*Ethics* Scientists may hold a view that a finding is not real until peer-reviewed. This perspective may lead them to delay communication of findings until after peer review. Our contention is that communicating provisional information with appropriate caveats is not inconsistent with an adherence to the role of peer review in scientific progress. Moreover, the desire to safeguard the scientific process must be weighed against the consequences of delay. For example, every day that a region with a species infected by a novel pathogen remains open to normal activities is a day when a pathogen can spread and the situation can quickly become unmanageable. Managers are able to take prudent actions to prevent spread while awaiting verification of findings and without compromising the rigor of the scientific process. We see no ethical basis to delay communication.

*Fear of being “scooped”* A final reason why a scientist might hesitate to communicate a finding with authorities is concern that someone else will publish the finding in a journal before they do. In practice, we suspect it to be unusual that selective communication with management would really raise this concern unless the scientist does not intend to publish for a long time. If it does arise, a scientist will have to weigh this concern against the ecological or societal cost of delay.

Ultimately, scientists must use their own judgement to decide when and how to communicate results. It may not always be clear when early communication of provisional findings is necessary or desirable, but our hope is that a discussion of these issues will alleviate some concerns that might otherwise delay appropriate action. While many applied scientists are experienced with communication of unpublished information, others may not have encountered a situation where their findings have consequences that require immediate attention. In the case of a novel pathogen, effective response relies on the rapid communication of a provisional discovery. In other cases, a provisional finding may not have the same level of urgency but selective communication about that finding could save someone a lot of effort. For example, a provisional taxonomic finding might have implications for relevancy of endangered species laws and knowledge of the finding could save individuals or institutions significant effort even if they must await peer review to fully act upon the information. In our view, communication of provisional findings that is clear about uncertainty and directed toward those who have relevant management authority can be considered whenever a scientist perceives that those authorities have a need to know.
